# Diagnostic yield of CT pulmonary angiography for pulmonary embolism in clinically suspected patients

**DOI:** 10.1097/MD.0000000000026213

**Published:** 2021-06-04

**Authors:** Ghazi Alshumrani, Ali Al bshabshe, Wesam Faried Mousa

**Affiliations:** a Department of Medicine (Radiology Division); b Department of Medicine (Adult Critical Care Division), College of Medicine, King Khalid University, Abha; c Department of Critical Care, Khamis General Hospital, Khamis Mushyet, Saudi Arabia.

**Keywords:** computed tomography, computed tomography pulmonary angiography, pulmonary angiography, pulmonary embolism, wells score

## Abstract

Pulmonary embolism (PE) is a common medical problem. Its diagnostic criteria must be reviewed to determine the need for confirmatory testing. Computed tomography pulmonary angiography (CTPA) is the current standard of care, which provides accurate diagnosis with rapid turnaround. This study aimed to estimate the diagnostic yield of CTPA in clinically suspected PE patients in a tertiary care hospital in Saudi Arabia.

Radiology records of all patients with clinically suspected PE who underwent CTPA between January 1, 2012 and September 30, 2018 were reviewed retrospectively. A radiologist with 10 years of professional experience interpreted and reported all cases. The Wells score with 2 tiers (likely and unlikely) was used to raise the clinical suspicion of PE.

Positive results for PE were reported in 177 out of 534 clinically suspected cases (33%). Among the positive PE cases, 143 were acute (81%) and 34 (19%) were chronic. Bilateral, right-sided, and left-sided PE were found in 115 (65%), 37 (21%), and 25 (14%) cases, respectively. Involvement of the segmental branches, subsegmental branches, and the pulmonary trunk were noted in 152 (86%), 70 (40%), and 9 cases (5%), respectively. Saddle PE was found in (4%) of the cases. The lower lobe branches (right 55%, left 53%) and the upper lobe branches (right 47%, left 41%) were the most common sites of involvement.

CTPA had a higher positive detection rate for PE among clinically suspected cases than its published diagnostic yield. Adequate clinical evaluation when selecting patients for CTPA is emphasized to minimize unjustified exposure of the patients to radiation and intravenous contrast administration. It is crucial for radiologists to provide detailed reports commenting on all relevant findings, including pertinent negatives. A template for reporting radiological findings for CTPA can be recommended for this purpose.

## 1 Introduction

Pulmonary embolism (PE) is the blockage of a pulmonary artery due to the movement of a substance through the bloodstream. It causes deprivation of blood flow that may lead to low blood oxygen levels with possible organ damage. A large PE or multiple clots can be a serious and fatal condition. Therefore, effective stratification of suspected PE in patients is required. Diagnostic computed tomography pulmonary angiography (CTPA) is the first choice when making a definitive diagnosis of PE.

CTPA carries the risk of radiation exposure and possible adverse effects of intravenous contrast administration. Therefore, an initial adequate clinical evaluation is crucial to raise a reasonable clinical suspicion in order to justify these potential risks. The study aimed to utilize the Wells score, a clinical diagnostic tool that is rarely evaluated in large clinical studies, and correlate its findings with CTPA results. Previous studies evaluated the diagnostic yield of CTPA in clinically suspected PE patients, but the utilization of Wells score was either not done or poorly documented.^[1,2]^

This retrospective study was designed to investigate the association between clinically suspected PE based on Wells score and CTPA results. The radiological findings of CTPA in all clinically suspected cases were listed and discussed.

## 2 Methods

After receiving approval from the regional ethics committee of King Khalid University (REC# June 5, 2018), we retrospectively evaluated the radiological records of all clinically suspected PE patients who underwent CTPA between January 1, 2012 and September 30, 2018 at a tertiary care hospital in the Southern region of Saudi Arabia.

Wells score with 2 tiers, likely ≥4.5 (consider CTPA) and unlikely ≤4, was used to raise the clinical suspicion of PE. The criteria of Wells score are: clinical signs and symptoms of DVT = 3, an alternative diagnosis is less likely than PE = 3, heart rate more than 100 = 1.5, immobilization for 3 or more consecutive days or surgery in the previous 4 weeks = 1.5, previous objectively diagnosed PE or DVT = 1.5, hemoptysis = 1, malignancy (on treatment, treatment in last 6 months or palliative) = 1.

All the scans were performed on a 64-slice CT scanner (Lightspeed VCT, General Electric, Milwaukee, WI), following standardized protocol. The scans were done in pulmonary arterial phase with 1.25 mm slice thickness reconstructed at 0.625 mm, kV of 100 to 120, and Auto mA. The administered intravenous contrast was Iobitridol (Xenetix, Guerbet, Roissy, France) 350 mg iodine/mL. The volume of contrast was 50 to 70 mL at a rate of 5 mL/second, followed by 20 mL normal saline at the same rate. Post processing multiplanar reconstruction was done on a dedicated workstation (Advantage Window, General Electric, Milwaukie, WI). A board-certified radiologist with ten years of professional experience interpreted the CTPAs for PE and other radiological findings. The quality of scans was assessed using a 5-grade scale (1 being poor quality scan, and 5 being excellent quality).

**Figure 1:**
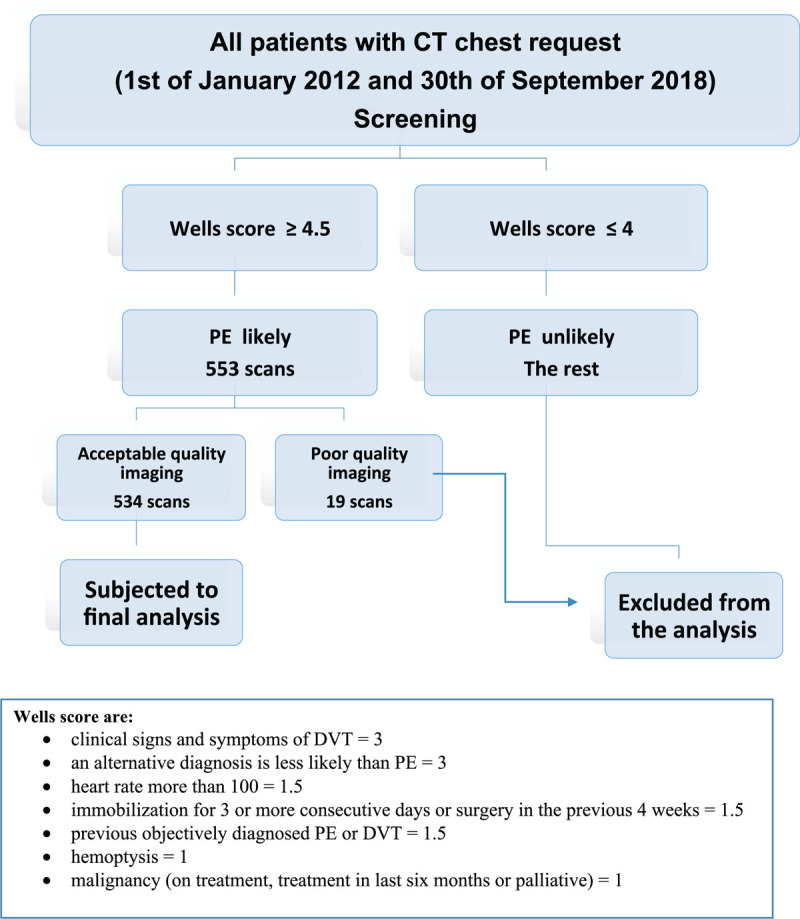
Flow chart of study methodology.

All patients with clinically suspected PE who had a Wells score of ≥4.5 were included in the study. Patients with a Wells score of ≤4, unavailable scores, or poor quality CTPA scans (1 out of 5) were excluded Figure 1.

### 2.1 Statistical analysis

An electronic data collection sheet was created to collate and code the data using a Microsoft Excel spreadsheet (Microsoft Corporation, Albuquerque, MM). The collected data were analyzed using Statistical Package for Social Sciences software (IBM Corp. Released 2016. IBM SPSS Statistics for Windows, Version 24.0. Armonk, NY). The percentage of different CPTA findings and the exact probability test (*P* value), which was considered to be significant when <.05, were calculated.

## 3 Results

We reviewed 553 CTPA scans of 266 males and 287 females. Quality of scanning was reported to be poor in 19 cases (3.44%), suboptimal in 145 cases (26.22%), acceptable in 169 cases (30.56%), good in 185 cases (33.45%), and excellent in 35 cases (6.33%). Poor quality scans were excluded from the study, leaving 534 scans with 256 and 278 males and females, respectively. There were no significant differences between the sexes (*P* < .05%) (Table 1).

**Table 1:** Patients scans demographic data.

Total CTPAs screened	553 scans		
Quality of the CTPA scans	Poor (excluded from the study)	19 scans	(3.44%)
	Suboptimal	145 scans	(26.22%)
	Acceptable	169 scans	(30.56%)
	Good	185 scans	(33.45%)
	Excellent	35 scans	(6.33%)
Included CTPAs	534 scans		
Gender	Males 256		
	Females 278		
Age	Mean	48.7	
	SD	20.2	
	min	18.0	
	max	103.0	
Scan results	Positive for PE	177 scans	(33.14%)
	Negative for PE	357 scans	(66.86%)
positive scans	Acute PE	143 scans	(81%)
	Chronic PE	34 scans	(19%)

Scan results were negative for PE in 357 cases (66.86%) of the clinically suspected patients, and positive in 177 cases (33.14%) (*P* < .01%). In positive scans, 143 cases were acute (81%), and 34 cases were chronic (19%) (*P* < .01%).

**Figure 2:**
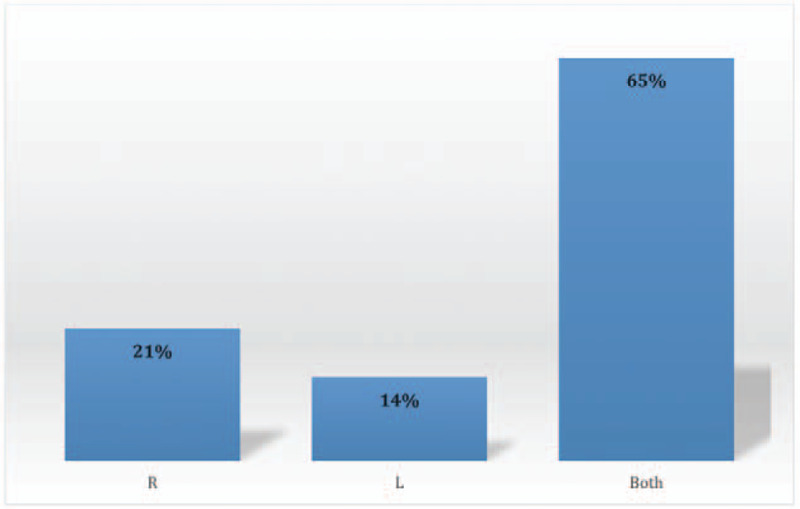
Distribution of positive PE cases by the side involvement.

**Figure 3:**
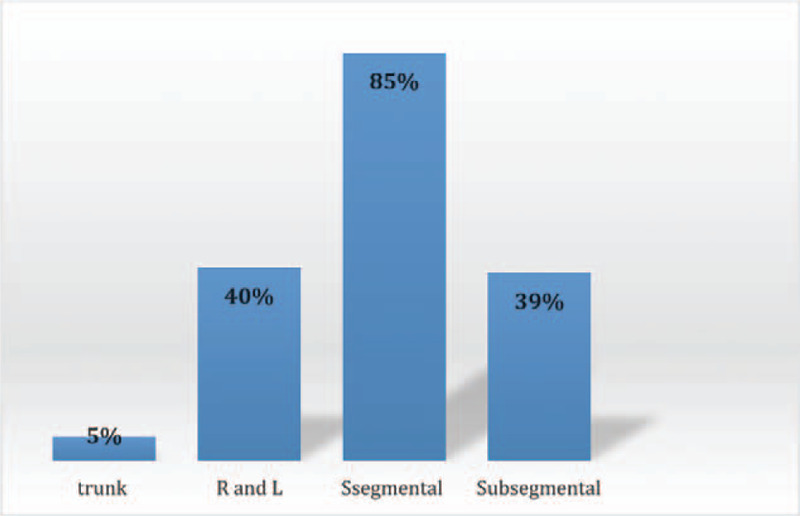
Distribution of positive cases by the pulmonary arteries site involvement.

**Figure 4:**
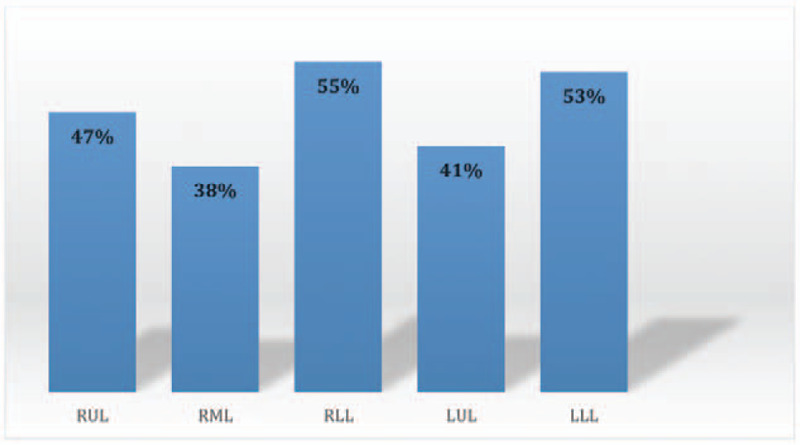
Distribution of positive PE cases by lobar branches involvement.

PE was detected in the right side in 37 cases (21%), the left side in 25 cases (14%), and bilaterally in 115 cases (65%) (Fig. 2). Involvement of the pulmonary trunk, right and/or left pulmonary arteries, segmental branches, and subsegmental branches were found in 9 (5%), 72 (41%), 152 (86%), and 70 (40%) cases (Fig. 3). Saddle PE (embolism that straddles the pulmonary trunk at its bifurcation) was reported in 7 cases (4%). In terms of the lobar distribution of pulmonary emboli, the most commonly involved sites were the lower lobe branches (right 55%, left 53%), followed by the upper lobe branches (right 47%, left 41%). The right middle lobe branches were the least involved and found in 38% of the cases (Fig. 4).

**Figure 5:**
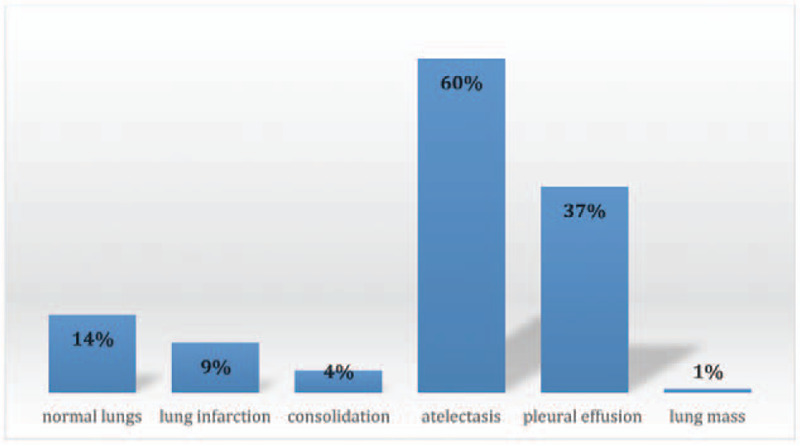
Lung findings in positive PE cases.

Other pulmonary findings in positive scans included atelectasis in 107 cases (60%), completely normal lungs in 24 cases (14%), lung infarction in 16 cases (9%), consolidation in 8 cases (4%), and incidental lung mass in one case (0.56%). Pleural effusion was reported in 67 cases (37%) (Fig. 5).

Other reported findings in positive PE cases included right ventricular strain (1.69%), air embolism (1.13%), pulmonary trunk diameter more than 3 cm (2.26%), and left ventricular thrombus (0.56%).

## 4 Discussion

CTPA data analysis of our study showed positive PE results in 33% of clinically suspected cases. In a recent systematic review ^[3]^ that included 12 studies, the diagnostic yield of CTPA for PE was shown to vary from 4.7% to 31%. Similarly, in a more recent prospective trial^[4]^ that included 1814 patients from 12 hospitals, the diagnostic yield of CTPA for PE was shown to be 25%. In another retrospective study^[5]^ done in a Canadian academic tertiary care center, a 15.9% positive rate for PE detection via CTPA was reported. In contrast to these previous studies, our study demonstrated a higher diagnostic yield of CTPA for PE. One possible cause for this (although this does not appear significant) is the exclusion of the patients with poor quality scans, which accounted only for 3.44% of the total cases. Utilization of the Wells score to raise the clinical suspicion for PE in our study is another likely cause of the high diagnostic yield. Low diagnostic yield of CTPA was suggested by multiple authors to reflect concerns for malpractice litigation.^[6,7]^ Also, it may reflect different clinical diagnostic algorithms when suspecting PE. More lenient clinical criteria may lead to the overuse of CTPA scans. The low diagnostic yield of CTPA for PE raises the need to criticize the criteria and/or investigations required for clinical suspicion of PE. Unnecessary CTPAs are expensive and carry the risks of radiation exposure and intravenous contrast administration.

As per hospital protocols, D-dimer levels were not included in our criteria for suspecting PE. Despite this, a higher percentage of positive scans was still achieved.^[8,9]^ Plasma D-dimer levels are elevated in acute thrombosis due to concurrent stimulation of coagulation and fibrinolysis.^[10]^ The negative predictive value of D-dimer testing is high, and a normal D-dimer level renders acute thrombosis unlikely. Conversely, the positive predictive value of elevated D-dimer levels is low and a high D-dimer is not useful for confirmation of acute thrombosis.^[11]^ D-dimer is more frequently elevated in patients with cancer, severe infection, inflammatory disease, and during pregnancy.^[12]^

Lung infarction occurs when an artery to the lung is blocked and part of the lung atrophies. It is most often caused by PE. However, the incidence of developing lung infarction in PE is low due to the dual blood supply to the lungs. Blood supply to the lungs comes from the bronchial circulation and the pulmonary circulation, so the tissue is more resistant to infarction.^[13,14]^ In our study, lung infarction was observed in a low percentage (9%) of cases. It is estimated that 16.9% of pulmonary emboli develop a small pulmonary infarction.^[15]^ Another study^[16]^ observed infarction in 29.2% of cases and proposed that the vast peripheral locality of the embolus beyond the anastomoses between the pulmonary and bronchial arteries may be crucial to determine the occurrence of an infarction. However, the percentage increases to 30% in large emboli^[15]^ and 60% in lethal PE.^[16,17]^ The low percentage in our results may be related to the differences in patient demographics across studies. In the acute setting, distinguishing ischemic pulmonary hemorrhage from lung infarction is difficult radiologically. However, hemorrhage without infarction usually resolves within a few days, while infarction evolves over months to lung parenchymal scarring.^[14,18]^

The incidence of pleural effusion in our study was shown to be 37% which was in accordance with the results shown in a previous systematic review of 19% to 61%.^[19]^ In a recent study,^[20]^ PE was associated with pleural effusion in around one-third of the patients and it was mainly small and bilateral. The emboli associated with pleural effusion in their study were mainly peripheral and usually associated with consolidation. However, clinically, these patients are characterized by an apparent discrepancy between the volume of the effusion, which is often not very large, and the severe accompanying dyspnea.^[21]^ The frequency of pleural effusion in PE is correlated with the severity of the embolism and with the occurrence of pulmonary infarction.^[21]^ The effusion is usually exudative in nature where the inflammatory mediators released from the pulmonary thrombi cause an increase in capillary permeability and the resulting interstitial edema fluid goes to the pleural space.^[22]^ Sometimes, it is a transudate where the increase in systemic venous pressure caused by PE increases hydrostatic pressure in capillaries leading to the accumulation of fluid in the pleural space.^[19]^

In our study, saddle PE was seen in 4% of cases. This was consistent with a previous study that showed an incidence of saddle PE of 2.6%.^[23]^ Saddle PE is a life-threatening condition that refers to a large thrombus that bestrides the bifurcation of the pulmonary trunk, extending to the left and right pulmonary arteries.^[24]^ In saddle PE, the patient may not be able to describe how he/she feels. Patients clinically present with cyanosis and agitation, with significant hemodynamic instability and a high potential for collapse. A high index of suspicion allows for early recognition and diagnosis via CTPA imaging, resulting in prompt initiation of the treatment.^[25]^

Basal atelectasis of the lungs following PE is due to the deterioration of gas exchange and lung mechanics distal to PE as well as surfactant system alterations. In our study, atelectasis was found in 60% of cases which agreed with previous clinical trials.^[26]^

Right ventricle (RV) strain was reported only in 1.69% of cases in our study. This finding is significantly lower to that of a previous study^[27]^ that noted RV strain in 72% of cases. This may have been caused by the underreporting of RV strain findings by the radiologist. Most of the time, the findings are not stated unless they are obvious or observed in the research settings. Echocardiography is more specific for reporting RV strain, so physicians have relied on this test more than CT, which is sensitive but less specific.^[27,28]^ In addition, echocardiography provides a dynamic real-time image that allows fine-tuning of views for better interpretation.^[29]^ Moreover, echocardiography confers additional positive prognostic value compared to CT in predicting post-PE clinical deterioration.^[27,30]^

Incidental lung mass was found in a very small percentage (0.56%) in our study. Malignant lung tumors are often accompanied by complications, such as venous thrombosis and PE. However, there is limited data regarding the clinical course of PE in lung cancer patients.^[31]^

In our study, a pulmonary trunk diameter of more than 3 cm was reported in 2.26% of cases. A normal diameter is less than 29 mm for men and less than 27 mm for women.^[32]^ A diameter of 3.32 cm has been shown to have a 58% sensitivity and 95% specificity for the presence of pulmonary hypertension.^[33]^ Chronic thromboembolic pulmonary hypertension results from the persistent obstruction of the pulmonary arteries due to acute or recurrent pulmonary emboli.^[34]^

Although CTPA is the method of choice for pulmonary vasculature imaging in patients with suspected PE, its observed sensitivity and specificity in PE diagnosis (for mainly 4-detector CT scanners) were 83% and 96%, respectively.^[35]^ Therefore, clinicians should consider further testing when clinical judgment and the CTPA result are not concordant.^[36]^ Subsegmental emboli were identified in 40% of the positive cases in our study. CTPA has a good interobserver agreement for diagnosing large and central PE. However, there may be significant discordance (25%) among radiologists when assessing small subsegmental contrast filling defects.^[37]^

Our results are adequately validated since all CTPA studies were reported by a single experienced radiologist. Moreover, we excluded all poor quality CTPA cases from our study. However, those 2 factors can be considered as limitations of our study. The single-center, small sample size, and retrospective design of the study are other limitations.

## 5 Conclusion

We report the positive PE detection rate of CTPA among clinically suspected cases to be 33%, which is higher than the published diagnostic yield of 10% to 15%. The higher diagnostic yield in this study demonstrates the importance of clinical evaluation, particularly with the Wells score, prior to exposing patients to the potential risks of radiation and contrast administration in CTPA. Adequate clinical evaluation should be emphasized when selecting patients who will undergo CTPA to minimize the unjustified exposure to radiation and intravenous contrast administration.

Since many physicians are not well-trained in interpreting CTPA, it is crucial for radiologists to provide detailed reports by commenting on all relevant findings and RV strain, including pertinent negatives. A radiology reporting template for CTPA can be adopted for this purpose.

## Author contributions

**Conceptualization:** Ghazi Alshumrani, Ali Al bshabshe.

**Data curation:** Ghazi Alshumrani, Ali Al bshabshe.

**Formal analysis:** Ali Al bshabshe, Wesam Faried Mousa.

**Investigation:** Ali Al bshabshe.

**Methodology:** Ali Al bshabshe, Wesam Faried Mousa.

**Resources:** Ali Al bshabshe.

**Writing – original draft:** Ghazi Alshumrani, Wesam Faried Mousa.

**Writing – review & editing:** Ali Al bshabshe.
